# Infection of CD8+CD45RO+ Memory T-Cells by HIV-1 and Their Proliferative Response

**DOI:** 10.2174/1874613600802010043

**Published:** 2008-07-10

**Authors:** Naveed Gulzar, Sowyma Balasubramanian, Greg Harris, Jaime Sanchez-Dardon, Karen F.T. Copeland

**Affiliations:** 1 National HIV and Retrovirology Laboratories, Public Health Agency of Canada, Ottawa, Canada; 2 Department of Biochemistry, Microbiology and Immunology, University of Ottawa, Ottawa, Canada; 3 Ottawa Health Research Institute, Ottawa, Canada

**Keywords:** CD8+ T-Cell, HIV-1, memory, proliferation, subset

## Abstract

CD8+ T-cells are involved in controlling HIV-1 infection by eliminating infected cells and secreting soluble factors that inhibit viral replication. To investigate the mechanism and significance of infection of CD8+ T-cells by HIV-1 *in vitro*, we examined the susceptibility of these cells and their subsets to infection. CD8+ T-cells supported greater levels of replication with T-cell tropic strains of HIV-1, though viral production was lower than that observed in CD4+ T-cells. CD8+ T-cell infection was found to be productive through ELISA, RT-PCR and flow cytometric analyses. In addition, the CD8+CD45RO+ memory T-cell population supported higher levels of HIV-1 replication than CD8+CD45RA+ naïve T-cells. However, infection of CD8+CD45RO+ T-cells did not affect their proliferative response to the majority of mitogens tested. We conclude, with numerous lines of evidence detecting and measuring infection of CD8+ T-cells and their subsets, that this cellular target and potential reservoir may be central to HIV-1 pathogenesis.

## INTRODUCTION

The Human Immunodeficiency Virus (HIV)/Acquired Immune Deficiency Syndrome (AIDS) pandemic is of great concern to the global population and much effort and research is necessary to understand and treat the viral disease. Currently, there are over 40 million people worldwide living with HIV/AIDS. HIV-1 is the etiologic agent that is responsible for AIDS, which occurs during the latter stages of viral pathogenesis (approximately 8-10 years after the initial infection).

	A recent source of controversy exists over the nature of HIV-1 infection of CD8+ T-cells. CD8+ T-cells play a crucial role in the cell-mediated response of the immune system as these cells are able to recognize and eliminate cells infected with foreign pathogens through their Cytotoxic T-lymphocyte (CTL) abilities [1]. In addition to their CTL abilities, the CD8+ T-cell Non-cytotoxic Antiviral Response (CNAR) can produce and secrete a number of soluble HIV-1 inhibitory factors that can block intracellular viral replication in CD4+ T-cells and other cellular subsets [2]. While there is an early increase in the number and function of HIV-1 specific CTLs, it has been established that in chronic infection, the decline in the CD8+ T-cell response accompanies progression to symptomatic disease [3, 4]. The significance and importance of CD8+ T-cells in viraemic control was elucidated in studies with Long-Term Non-Progressors (LTNP).

CD8+ T-cells from LTNPs of HIV-1 infection maintain normal CD8+ T-cell numbers, greater functionality and persistent HIV-1 specific CTL responses than those of progressors [4, 5].

Although the central focus of HIV-1 pathogenesis has been on the role of CD4+ T-cells, there has been much recent evidence demonstrating the susceptibility of CD8+ T-cells to HIV-1 infection both **in vivo** and **in vitro**(reviewed in [1]). The cause of CD8+ T-cell dysfunction and depletion during HIV-1 infection **in vivo** still remains unclear. This phenomenon is generally attributed to the lack of CD4+ T-cell help or to the secretion of soluble viral proteins in the viral milieu. It has been demonstrated that the presence of CD4+ T-cells and antigen presenting cells are critical in CD8+ T-cell maturation and function [6]. In addition, antigenic stimulation of CD8+ T-cells may result in a state of anergy and possibly cell death [7] inducing an accompaniment of chronic unresponsiveness in the immune system. Work done by Potter *et al.* and others have implicated CD8+ T-cells as a reservoir of circulating HIV-1 infected cells [8, 9]. Direct infection of CD8+ T-cells may also explain the abnormalities and dysfunction seen in this subset during AIDS pathogenesis. Over the past couple of years, numerous mechanisms have been proposed to explain HIV-1 entry into CD8+ T-cells. One such mechanism involved the selection of CD8-tropic variants of HIV-1 that were syncytium inducing and cytopathic for CD8+ T-cells [10, 11]. A few of these viral isolates from HIV-1 infected individuals were shown to be able to use the CD8 cell-surface molecule as a receptor [11]. Cell-to-cell transfer of viral particles has also been postulated as a mode of HIV-1 spread and entry into CD8+ T-cells [12, 13]. Lastly, a number of investigators have shown that the expression of the CD4 molecule on CD8+ T-cells increased subsequent to activation [14-18]. These aptly termed CD4^dim^CD8^bright^ T-cells were found to be susceptible to HIV-1 infection. Key studies by Hughes *et al.* demonstrated that virus derived from HIV-1 infected CD8+ T-cells accounted for approximately 15% of the plasma viral load **in vivo**[19].

One of the hallmarks of the immune system is the ability to generate memory. This defining aspect of the immune response is critical in controlling and recognizing viral infections. In response to an infection, CD8+ T-cells differentiate. Upon antigen recognition through Major Histocompatibility Complex (MHC)-I molecules, naïve CD8+ T-cells (CD8+ CD45RA+CD28+CCR7+) become activated and differentiate into memory (CD8+CD45RO+CD28+CD27+CCR7+) and effector (CD8+CD45RA+CD28-CD27-CCR7-) T-cells [20]. Once the immune response subsides and the infection is cleared, the majority of the activated CD8+ T-cells are eliminated, but a few remain circulating in the immune system. These circulating cells exist as memory cells that are pathogen-specific [21]. In addition, the memory CD8+ T-cells are able to circulate for extended periods of time because of high levels of anti-apoptotic molecules [22] and, unlike naïve CD8+ T-cells, they are able to proliferate in the absence of antigen **in vivo**[23].

Impairment of the function and activities of CD8+ T-cells could allow the virus to escape from immune containment and further contribute to the deterioration of the infected individual’s health. Early studies examining the effects of HIV-1 infection of CD8+ T-cells noted that infection was minimal **in vivo**. However, these studies did not use very sensitive techniques to detect HIV-1 infection and the associated effects. More recent studies using sensitive quantitative RT-PCR and flow cytometry have demonstrated frequent infection of CD8+ T-cells. To investigate the susceptibility of CD8+ T-cells to HIV-1 infection *in vitro* and the functional significance of infection, we examined levels of HIV-1 replication and the effects of viral infection in CD8+ T-cells and T-cell subsets. The susceptibility of CD8+ T-cells to T-cell tropic and macrophage tropic strains of HIV-1 was also analyzed. In addition, we determined the frequency of HIV-1 infection in the CD8+ T-cell population as measured by an intracellular HIV-1 assay. Lastly, we examined the effects of HIV-1 infection on CD8+ T-cell subsets. We hypothesized that CD8+ T-cells would be able to support productive HIV-1 replication and may serve a role as a cellular reservoir for HIV-1 production and replication. From our results, we observed that CD8+ T-cells were able to support productive infection with T-cell tropic viral strains independent of CD4 cell-surface expression. Interestingly, HIV-1 replicated preferentially in the CD8+ T-cell memory subset, resulting in an increase in the activation state of these cells. However, the infection-induced activation did not alter the ability of the memory T-cells to proliferate in response to the majority of mitogens used for stimulation. The identification of a potential novel cellular target and reservoir may have a profound effect on our views of HIV-1 disease progression and treatment.

## MATERIALS AND METHODOLOGY

### Isolation of Lymphocyte Populations

Blood samples from healthy volunteers were collected into tubes containing Ethylenediamine Tetra-acetic Acid (EDTA) by venipuncture. Samples were diluted with an equal volume of Phosphate Buffered Saline (PBS) and Peripheral Blood Mononuclear Cells (PBMCs) were isolated by Ficoll-Hypaque (Amersham Biosciences, Baie d’Urfe, PQ) density centrifugation. Subsequent to density centrifugation, isolated PBMCs were washed twice in PBS. CD8+ and CD4+ T-cell populations were isolated from freshly purified PBMCs through positive selection using anti-CD8 and -CD4 conjugated magnetic beads (Miltenyi, Auburn, CA) respectively, according to the manufacturer’s protocol. Briefly, cells were labeled with the magnetically conjugated antibody of interest for 20 minutes at 4ºC. Subsequent to labeling, cells were placed through a column inserted in the magnetic field of a MACS separator (Miltenyi) and unbound cells were washed with MACS buffer (PBS, 0.5% Bovine Serum Albumin (BSA), 2 mM EDTA, pH 7.2). After washing unbound cells, the column was removed from the separator and the labeled cells were eluted with the addition of MACS buffer. Following elution, the isolated T-cell subsets were washed twice with PBS.

The isolation of CD8+ T-cell subsets was performed using anti-CD8 Multisort conjugated magnetic beads (Miltenyi). In order to isolate the T-cell subsets, a second round of positive selection with magnetic bead-conjugated antibodies to either CD45RO+ or CD45RA+ was performed. Similarly, CD28, CD38 and HLA-DR positive and negative populations were selected using the Multisort conjugated magnetic beads.

### Cell Culture

PBMCs and T-cell subsets were cultured in RPMI-10 (RPMI 1640 medium supplemented with 10% Fetal Calf Serum (FCS), 100 U/mL penicillin (Invitrogen, Burlington, ON), 100 μg/mL streptomycin (Invitrogen), and 20 U/mL Interleukin (IL)-2 (Sigma-Aldrich, St. Louis, MO)). Cells were incubated at 37(C with 5% CO_2_.

### Sources of Viruses

The T-cell tropic laboratory strain HIV_IIIB_ was obtained from Advanced Biotechnologies Incorporated (Columbia, MD). The macrophage tropic laboratory strain, HIV_ADA_, was obtained from the National Institutes of Health (NIH) AIDS Research and Reference Reagent Program (Germantown, MD). The clinical isolates of HIV-1 were generated from the blood of HIV-1 infected individuals. Briefly, PBMCs were isolated from the peripheral blood of infected subjects by Ficoll-Hypaque density centrifugation. The CD8+ T-cell fraction was removed from the isolated PBMCs through the use of anti-CD8 conjugated magnetic microbeads. The remaining cells were subsequently cultured with HIV-negative PHA blastocytes in RPMI-10. The cultures were monitored at days 10 and 15 for HIV-1 p24 production by an Enzyme-Linked Immunosorbent Assay (ELISA) (SAIC-Frederick Inc., Frederick, MD).

The titre of the viral stock was determined by a TCID_50_ assay. Briefly, 100 µL serial dilutions of the viral stock (approximately 1,500 ng/mL as determined by a HIV-1 p24 ELISA) was used to infect 250,000 MT2 cells (American Type Culture Collection (ATCC), Manassas, VA) in quadruplicate in a 96-well flat bottom plate. Subsequent monitoring for the formation of syncytia was performed over a 7-day culture period. The titre of the viral stock was determined by the Reed-Muench accumulative method. The titre of the HIV_IIIB_ viral stock used for CD8+ T-cell infections was found to be 1 x 10^5^. Unless otherwise noted, 300 TCID_50_/mL of the viral inoculum for each of the viral isolates was used to infect isolated cells at a density of 1 x 10^6^ cells/mL during the course of the *in vitro* experiments.

### HIV-1 Infection and Monitoring of Replication in CD8+ T-Cell Populations

Immediately after isolation and prior to infection, CD8+ T-cells were stimulated with 2.5 μg/mL of Phytohemagglutinin (PHA) (Sigma-Aldrich) for three hours followed by subsequent washes and a 1 hour incubation with 2 μg/mL polybrene (Sigma-Aldrich) in RPMI-10. Subsequently, the cells were washed, re-cultured in RPMI-10 and infected with 300 TCID_50_/mL of the appropriate viral strains and isolates. Three days after the infection, the cells were extensively washed to remove any extracellular virus and debris and were re-cultured in RPMI-10 and this time point was considered the zero time point in the infection assays. In order to determine the levels of HIV-1 replication and production, cell-free supernatants were taken at days 5, 10 and 15 (or as indicated in the results and figures) post-infection from the infected cell cultures. Virus replication was assessed by HIV-1 production as measured by an ELISA (SAIC-Frederick Inc.). In order to normalize our results, supernatants were taken from cultures of CD8+ T-cells derived from several different individuals.

	In order to assess productive infection, the reverse transcriptase inhibitor, Azidothymidine (AZT) (NIH AIDS Research and Reference Reagent Program) was used to treat the cells at a concentration of 10 μM for one hour prior to infection and was not used further throughout the time course.

### Nucleic Acid Extraction and Detection of Productive HIV-1 Infection by RT-PCR Analysis

Total RNA was isolated from 3 x 10^6^ cells using the GeneElute Mammalian Total RNA Miniprep Kit (Sigma-Aldrich). First strand cDNA was reverse transcribed with a pd(N)_6_ primer and Moloney Murine Leukaemia Virus (MMLV) reverse transcriptase using the First-Strand cDNA synthesis kit (Amersham Biosciences) according to the manufacturer’s protocol. RT-PCR reactions were set up for each cDNA towards the *gag* region of HIV-1 (p24 forward: 5’-ATAGAGGAAGAGCAAAACAAAA-3’ p24 reverse: 5’-CAAAATTACCCTATAGTGCA-3’). Each reaction was conducted in a total volume of 25 µL containing 2.5 µL of 2 mM dNTP (Amersham Biosciences), 1.25 µL of 50 mM MgCl_2_ (Amersham Biosciences), 1 µL of each primer (from a 100 mM stock) and 1 U of Taq DNA polymerase (Amersham Biosciences). A total of 5 µL of each cDNA was used in the RT-PCR analysis. RT-PCR amplification was performed for 35 cycles of: denaturing at 94ºC for 60 seconds, annealing at 55ºC for 60 seconds and extension at 72ºC for 60 seconds. This was followed by a final extension for 10 minutes at 72ºC. RT-PCR amplicons were run at 80 V on a 1% agarose gel containing 0.5 µg/mL of ethidium bromide and visualized under Ultraviolet (UV) light.

### Flow Cytometric Analysis

Flow cytometric analysis was gated on the live cell populations. Measurement of the levels of intracellular HIV-1 staining was performed as follows: 1 x 10^6^ cells were permeabilized with the Fix &amp; Perm cell permeabilization kit (Caltag Laboratories, Burlingame, CA) according to the manufacturer’s protocol. Cells were labeled with 10 µL of the appropriate fluorochrome-conjugated monoclonal antibodies CD8-PC5 (Serotec, Raleigh, NC), CD4-PC5 (Serotec) and HIV-1_gag_-FITC (Coulter clone KC57, Beckman Coulter, Fullerton, CA) prior to flow cytometry analysis. The KC57 antibody identifies HIV-1 core antigens (p55, p39, p33 and p24). Results were assessed on an Epics ALTRA system (Beckman Coulter) after gating on lymphocytes based on their forward and side scatter properties. Analysis was performed using Expo32 software (Applied Cytometry Systems, Sacramento, CA) and was based on a minimum of 10, 000 events. Values are expressed as the mean fluorescence intensity.

For the analysis of the purity of the isolated populations of CD8+ T-cells, the following fluorochrome-conjugated monoclonal antibodies were used: CD8-PE (Serotec), CD4-FITC (Serotec), CXCR4-PE (BD Biosciences, San Diego, CA), CCR5-PE (BD Biosciences), IgG-FITC (BD Biosciences), CD3-FITC (BD Biosciences), CD56/16-PE (BD Biosciences). For flow cytometric analysis, the cells of interest were washed twice in PBS and once in flow binding buffer (PBS, 0.1% NaN_3_, 2% BSA). The cells were subsequently resuspended at a concentration of 1 x 10^6^ cells per 100 μL of flow resuspension buffer and were labeled with 10 μL of the appropriate antibody for 30 minutes at room temperature. Prior to flow cytometric analysis, the cells were fixed in 200 μL of PBS/4% paraformaldehyde at 4ºC for 5 minutes. Cell-surface molecule expression during the course of HIV-1 infection was examined in positively selected cells using CD8-PE (Serotec), CD4-FITC (Serotec), CXCR4-PE (BD Biosciences), CCR5-PE (BD Biosciences), CD45RA-PE (BD Biosciences),CD45RO-FITC (Immunotech, Fullerton, CA), CD28-FITC (BD Biosciences), CD38-FITC (BD Biosciences) and HLA-DR-FITC (BD Biosciences) conjugated monoclonal antibodies. In order to determine the surface expression of both CD45RA and CD45RO in the CD8+CD4- and CD8+CD4+ populations, cells were examined by flow cytometry at day 5 post-infection after staining with antibodies directly conjugated to FITC.

### Lymphocyte Proliferation Assay

Lymphocyte proliferation was measured by the XTT based colorimetric assay (Roche Laboratories, Meylan, France). In the memory CD8+ T-cell studies, CD8+CD45RO+ T-cells were positively isolated from the PBMCs of a healthy volunteer by magnetic labeling. Cells were either left uninfected or infected with 300 TCID_50_/mL of HIV_IIIB_. For proliferation tests, approximately 450,000 cells were seeded in triplicate in 100 μL of culture medium (RPMI-10 supplemented with 20 U/mL of IL-2) per well of a 96-well flat bottom plate. Cells were incubated in the presence of each of the following mitogens: 3 µg/mL αCD3/CD28 (Sigma-Aldrich); 10 ng/mL Phorbol Myristate Acetate (PMA) (Sigma-Aldrich); 2 U/mL Tetanus Toxoid (TT) (Sigma-Aldrich); and 5 µg/mL PHA. On days 2-3 post-mitogenic stimulation, cells were labeled with 50 μL of the XTT labeling reagent as per the manufacturer’s protocol. The proliferation index was assessed by measuring the absorbance at 492 nm (with a reference wavelength at 690 nm) 18 hours post-labeling.

### Statistical Analysis

Data are expressed as the mean ± SEM. Differences in measured variables between experimental and control groups were assessedusing the Student’s *t* test. Statistical significance was accepted at *p* < 0.05.

## RESULTS

### Susceptibility of Primary Blood-Derived CD8+ T-Cells to HIV-1 Infection *in vitro*

**Fig. (1):**
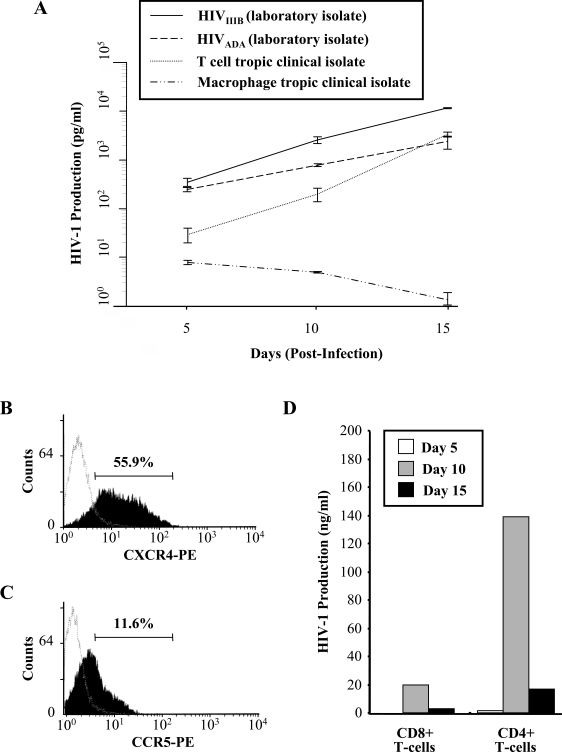
Infection of CD8+ T-cells by HIV-1. (A) Selected CD8+ T-cells were infected with 300 TCID_50_/mL of the T-cell tropic and macrophage tropic laboratory strains and clinical isolates of HIV-1. HIV-1 p24 production was measured by an ELISA at the indicated time points with cell-free supernatants from cultures. Results show the mean HIV-1 production and SEM of three independent experiments. (B) CXCR4 receptor expression on CD8+ T-cells as measured by flow cytometry. One representative of three experiments is shown. The mean fluores-cence intensity of CXCR4 receptor and SEM of three independent experiments is 55.9% ± 2.1. (C) CCR5 receptor expression on CD8+ T-cells. One representative of three experiments is shown. The mean fluorescence intensity of CCR5 receptor and SEM of three independent experiments is 11.6 ± 1.0. CD8+ T-cells were also stained with negative controls (isotype control, dashed line). (D) Positively selected CD4+ and CD8+ T-cells were infected with 300 TCID_50_/mL of HIV_IIIB_. HIV-1 production was measured by an ELISA at the indicated time points from cell-free supernatants

After establishing the purity of the selected CD8+ T-cell population and demonstrating the absence of any contaminating CD4+ T-cells or Natural Killer (NK) cells (data not shown), the ability of CD8+ T-cells to support HIV-1 infection was ascertained. *in vitro* infection of primary CD8+ T-cells was performed with T-cell and macrophage tropic strains of HIV-1, along with Non-Syncytia Inducing (NSI) and Syncytia Inducing (SI) clinical isolates of the virus. The CD8+ T-cells were infected with an equal amount of virus, allowing the infection sensitivity of the CD8+ T-cells for the viral isolates and strains to be compared. Fig. (**1A**) shows a 15 day time course of virus replication of the two laboratory strains and the two clinical isolates of HIV-1 in positively selected CD8+ T-cells. As measured by a HIV-1 p24 ELISA, the T-cell tropic strain, HIV_IIIB_, maintained the highest levels of viral production in the CD8+ T-cell population and the levels of virus replication for the HIV_IIIB _strain reached a peak at day 15 post-infection (Fig. **1A**). The replication of the T-cell tropic clinical isolate was also supported in CD8+ T-cells, although to lower levels than the laboratory strain. Similarly, CD8+ T-cells also supported moderate levels of replication of the macrophage tropic laboratory strain, HIV_ADA_, but replication of the macrophage tropic clinical isolate was poor.

In contrast to the viral kinetics of the T-cell and macrophage tropic laboratory strains and the T-cell tropic clinical isolate, the levels of HIV-1 production for the macrophage tropic clinical isolate declined over the 15 day time course. Input virus was not the cause of the low levels of viral production observed by this clinical isolate as CD8+ T-cells were extensively washed 3 days post-infection and day 0 of the infection assay was denoted at this point. Based on the above results, the HIV_IIIB _strain was used in subsequent experiments due to the ability of CD8+ T-cells to support high levels of replication of this virus.

Most HIV-1 strains require CXCR4 or CCR5 as a co-receptor for viral entry, along with CD4 expression. The surface expression of the chemokine receptors on primary CD8+ T-cells was examined by flow cytometry. It was observed that the CXCR4 chemokine receptor was significantly expressed on the surface of the CD8+ T-cells (55.9% ± 2.1, Fig. 1B). The expression of the CCR5 chemokine receptor was only moderately expressed on the CD8+ T-cell-surface (11.6% ± 1.0, Fig. 1C). Comparison of viral replication kinetics was assessed between CD8+ and CD4+ T-cells over a 15 day time course (Fig. **1D**). CD8+ T-cells were found to support approximately 10-fold lower levels of HIV-1 replication than CD4+ T-cells *in vitro*. Fig. (**1D** &amp **1Aa**) illustrate differences in CD8+ T-cell infection and replication kinetics of viral production. A caveat of *in vitro* infection is the observation of these variances due to differences in CD8+ T-cell populations used for the studies and the requirement for cell culture activation through cytokines/antigens for productive infection.

**Fig. (2):**
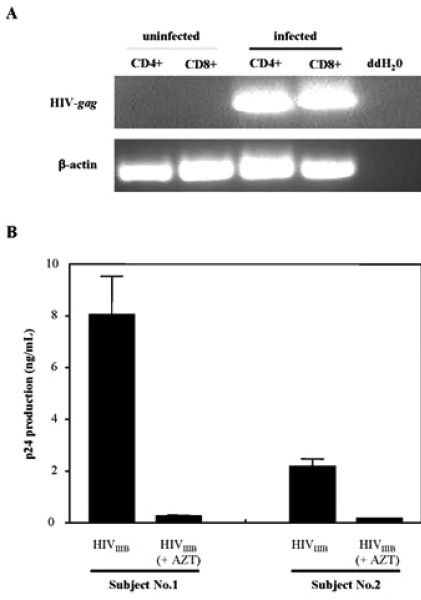
Productive HIV-1 infection of CD8+ T-cells *in vitro*. (A) RT-PCR analysis of HIV-gag transcripts in the uninfected and infected populations of isolated CD4+ and CD8+ T-cells. Isolated lymphocyte populations were left uninfected or infected with 300 TCID_50_/mL of HIV_IIIB_ for 7 days prior to RT-PCR analysis. Detection of β-actin was run as a loading control. Parallel samples were run without reverse transcriptase as a control for DNA contamination of the samples (data not shown). (B) Infection of CD8+ T-cells is sensitive to AZT. Infection was performed in the absence and presence of AZT and HIV-1 production was measured by an ELISA for the HIV-1 gag protein, p24. Cells were treated with 10 µM of AZT for one hour prior to infection. CD8+ T-cells from two uninfected individuals were infected with 300 TCID_50_/mL of HIV_IIIB_ laboratory strain. The results shown are the mean and SEM of three independent experiments.

Productive infection was demonstrated by RT-PCR analysis. Only HIV-1 infected CD8+ and CD4+ T-cells, showed that viral infection was productive as both T-cell populations showed a HIV-1 *gag*-specific band when RT-PCR analysis was performed (Fig. **2A**). Productive infection was also demonstrated in Fig. (**2B**) where the presence of AZT abrogated HIV-1 replication in CD8+ T-cells isolated from two volunteers. Differences in the levels of viral production were also observed between the two subjects. Subject 2 supported lower levels of HIV-1 production in their isolated CD8+ T-cells in the absence of AZT, indicating differences in viral replication supported by CD8+ T-cells from various individuals.

**Fig. (3):**
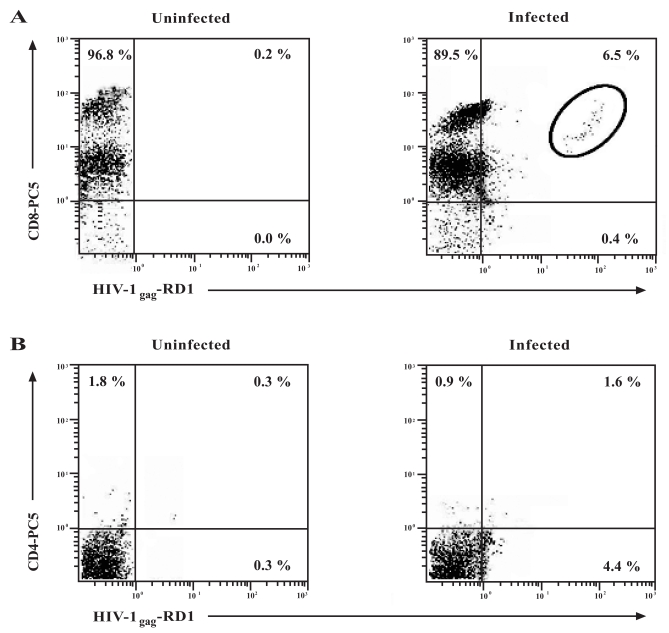
Detection of intracellular HIV-1 expression in CD8+ T-cells. CD8+ T-cells were infected with 300 TCID_50_/mL of HIV_IIIB_or left uninfected. (A) Intracellular p24 was examined on the CD8+ T-cell population by flow cytometry at day 15 post-infection with antibodies to HIV-1_gag_ (p24-RD1) and CD8. In the infected population, the number in the upper right quadrant represents the number of cells found in the circle as to minimize any false-positives. (B) To determine the contribution of CD4+ cells to *in vitro* CD8+ T-cell infection, intracellular HIV-1 expression was examined in the CD8+ T-cell population by flow cytometry at day 15 post-infection with antibodies to HIV-1_gag_ and CD4 only. Numbers in the quadrants represent the percentage of gated cells in each quadrant. A minimum of 10, 000 events was assessed for each analysis.

To further determine whether CD8+ T-cells could support HIV-1 infection, flow cytometry was performed to detect the presence of HIV-1 p24 (HIV-1_gag_) intracellularly in this population. As shown in Fig. (**3A**, circle, upper right quadrant), 6.5% of the total CD8+ T-cell population were infected intracellularly with HIV-1_gag_. As a control for the experiment, the contribution of CD8+CD4+ cells to the 6.5% of total infected cells was determined. The isolated and infected CD8+ T-cells were stained additionally with a CD4 antibody and intracellular HIV-1 expression was again measured by flow cytometry (Fig. **3B**). From the results, approximately 1.6% of the CD8+CD4+ cells contributed to the total levels of the CD8+ T-cell infection obtained in Fig. (**3A**). They did not contribute significantly to the total levels of infection due to the low mean fluorescence intensity of this population. Two distinct populations of CD8+ T-cells were observed in the flow cytometric analysis based upon the expression levels of the CD8 molecule. These populations, that have been observed by others, differ in their surface expression of the CD8 cell-surface molecule and have been aptly termed CD8^high^ and CD8^low^ expressing populations [24, 25]. A similar experiment illustrated that approximately 20 - 25% of CD4+ T-cells stained intracellularly for HIV-1 (data not shown).

### Expression of the CD4 Cell-Surface Molecule on the Surface of CD8+ T-Cells During HIV-1 Infection

**Fig. (4):**
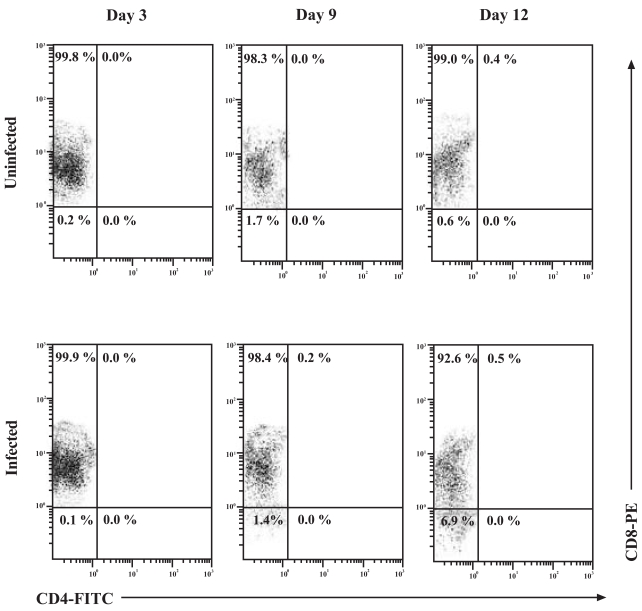
CD4 cell-surface molecule up-regulation on the surface of CD8+ T-cells. Uninfected and HIV-1 infected CD8+ T-cells were examined at the indicated time points by flow cytometry for the expression of the CD8 and CD4 cell-surface molecules. CD8+ T-cells were infected with 300 TCID_50_/mL of _IIIB_ and cell-surface expression of CD8 and CD4 was assessed at days 3, 9 and 12 by flow cytometry. Numbers in the quadrants represent the percentage of gated cells in each quadrant. A minimum of 10, 000 events were assessed for each analysis.

To examine the possibility of CD4 up-regulation in the *in vitro* infection system used throughout the studies, CD8+ T-cells were infected and monitored for CD4 expression by flow cytometry over a 12 day time course (Fig. **4**). Interpretation of the flow cytometry data showed very distinct populations. Analysis of both the uninfected and infected CD8+ T-cell populations showed that CD4 expression on these cells reached a maximum of 0.4% and 0.5%, respectively, on day 12 of culture. This suggests that cell culture conditions, not HIV-1 infection *per se*, resulted in this observed up-regulation of CD4 on the surface of CD8+ T-cells. The overall majority of CD8+ T-cells were of the single-positive phenotype and the contribution of Double Positive (DP) cells was found to be minimal.

### Memory CD8+ T-Cells Support Higher Levels of HIV-1 Replication

**Fig. (5):**
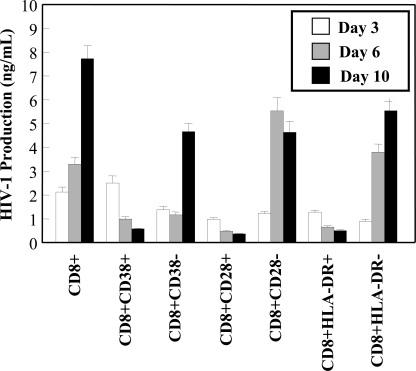
HIV-1 replication in CD8+ T-cell subsets. CD28, CD38 and HLA-DR positive and negative populations were infected with HIV-1 and cultured. Cell-free supernatant samples were taken post-infection at the indicated time points for measurements of HIV-1 production by an ELISA. Results are the mean and SEM of three independent experiments.

Subsets of CD8+ T-cells have been shown to be significant in the control of HIV-1 infection and disease progression. It has been postulated that higher levels of CD8+ CD38+ T-cells may be associated with poor prognosis and were found to be elevated in individuals who have progressed to AIDS [26, 27]. In contrast, high levels of CD8+HLA-DR+ cells were observed in asymptomatic individuals [28]. In studies with CD8+ CTLs, HLA-DR expression was found to correlate with proliferation and CD28 expression [29]. Based on the possibility that HIV-1 infection may be involved in hindering the function and activities of various key CD8+ T-cell subsets, the susceptibility of the aforementioned subsets to *in vitro* HIV-1 infection was examined. Viral production was measured in the CD8+ T-cell subset cultures over a 10 day time course by a HIV-1 p24 ELISA. The negative fractions obtained from the subset isolation were also assessed for HIV-1 production. Interestingly, the CD8+CD28+, CD8+CD38+ and CD8+HLA-DR+ subsets all supported very low levels of HIV-1 replication (Fig. **5A**). In fact, the levels of viral production decreased over the course of *in vitro* infection in these subsets. Fig. (**5A**) also showed that 2.5 -4 fold higher levels of replication were supported in the CD8+CD28-, CD8+CD38- and CD8+HLA-DR- populations, compared to the positive fractions.

**Fig. (6):**
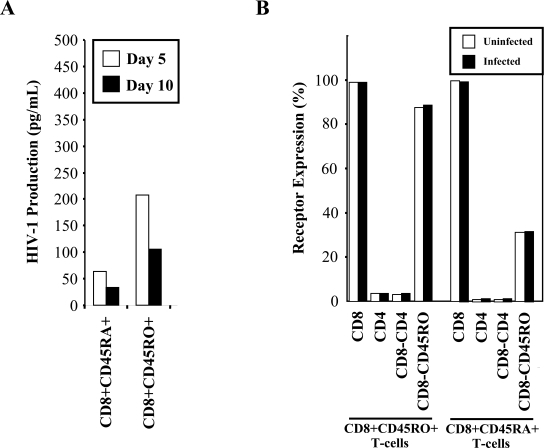
HIV-1 preferentially replicates in the CD8+CD45RO+ memory T-cells. (A) CD45RO positive and CD45RA positive populations were infected with 300 TCID_50_/mL of _IIIB_ and cultured. Supernatants were taken at days 5 and 10 post-infection for p24 measurement by ELISA. Results are representative of two independent experiments. (B) Uninfected (□) and infected (■) memory and naïve T-cells were ex-amined for CD45RO, CD45RA, CD4 and CD8 receptor expression by flow cytometry at day 5 post-infection.

Susceptibility of the memory and naïve CD8+ T-cell populations was also examined in response to HIV-1 infection with the HIV_IIIB _strain. The CD8+CD45RA- and CD8+CD45RO+ memory cell populations both supported approximately 5 – 10 times higher levels of HIV-1 replication than the CD8+CD45RA+ and CD8+CD45RO- naïve populations (Fig. **6A**). The p24 levels obtained in these experiments were lower than the levels found in previous experiments, as these infections were performed using lower levels of PHA stimulation to minimize any possible up-regulation of receptors. We examined receptor expression during the course of infection (Fig. **6B**) and confirmed by flow cytometry that there was no alteration in receptor expression during the course of *in vitro* infection within the memory and naïve CD8+ T-cell populations.

### Proliferation of Memory CD8+ T-Cells in Response to HIV-1 Infection

**Fig. (7):**
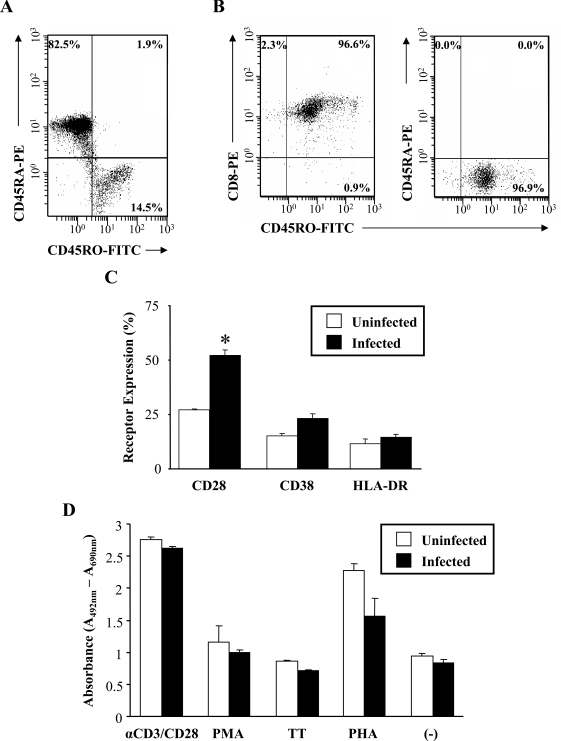
Proliferative response of HIV-1 infected CD8+CD45RO+ memory T-cells in response to antigenic stimulation. (A) Positively selected CD8+ T-cells were stained with CD45RO-FITC and CD45RA-PE to determine the percentage of memory cells in the isolated CD8+ T-cell population. (B) The purity of the selected CD8+CD45RO+ memory T-cells was assessed by flow cytometry and staining with CD45RO-FITC, CD45RA-PE (right histogram) and CD8-PE (left histogram). Numbers in the quadrants represent the percentage of gated cells in each quadrant. (C) Expression of the activation markers CD28, CD38 and HLA-DR in uninfected (□) and infected (■) CD8+CD45RO+ memory T-cells. Results are the mean percentage and SEM of three independent experiments as measured by flow cytometry. Statistical significance of p < 0.01 is denoted by an (*). (D) Lymphocyte proliferative ability of uninfected (□) and infected (■) CD8+CD45RO+ memory T-cells as measured by the XTT based colorimetric assay. Memory T-cells were infected (or left uninfected) for 7 days prior to antigen stimulation. Cells were stimulated with αCD3/CD28, Phytohemagglutinin (PHA), Phorbol Myristate Acetate (PMA), Tetanus Toxoid (TT) or left unstimulated (-). Proliferation was measured at days 2-3 post-antigenic stimulation by labeling with an XTT reagent during the last 18 hours of culture. Results are the mean and SEM of three independent experiments. Data is expressed as the mean absorbance (A_492nm_ – A_690nm_) and SEM.

HIV-1 infection has been reported to affect the frequency and function of circulating CD8+ T-cells, thereby hindering the ability of these cells to eradicate viral infection and maintain T-cell mediated immunity. In order to better define the effect of HIV-1 infection on the function and abilities of the CD8+ T-cell memory subset, CD8+CD45RO+ T-cell proliferation in response to stimulation was measured. As assessed by flow cytometry and shown in Fig. (**7A**), the majority of the isolated CD8+ T-cells were of the naive subset (82.5% CD8+CD45RA+ naive T-cells; 14.5% CD8+CD45RO+ memory T-cells). The purity of our selected CD8+CD45RO+ memory T-cell population was also assessed. The selected memory T-cell population was devoid of any contaminating CD8+CD45RA+ naïve T-cells (Fig. **7B**, right histogram) and was composed of approximately 97% pure cells of the CD8+CD45RO+ phenotype (Fig. **7B**, left histogram).

Prior to mitogen stimulation, the CD8+CD45RO+ memory T-cells were infected (or left uninfected) for 7 days. Before measuring proliferation, the activation state of the memory cells was assessed before and after infection (Fig. **7C**). Based upon the flow cytometry results, the mean fluorescence of CD28 expression nearly doubled (uninfected,

27.3% ± 0.3 infected, 52.3% ± 2.4) during the course of HIV-1 infection and this was found to be significant (p-value = 0.0026). There were marginal increases in CD38 and HLA-DR expression in response to infection. To determine the proliferative capacity of the memory subset, cells were stimulated for 2-3 days with various mitogens both in the presence and absence of HIV-1 infection. Based upon the results, it was found that minimal differences in *in vitro* lymphocyte proliferation between infected and uninfected CD8+CD45RO+ memory T-cells in response to mitogenic stimulation (Fig. **7D**) were observed. Overall, HIV-1 infection resulted in a slightly reduced rate of proliferation in the CD8+CD45RO+ memory subset. Mitogenic stimulation with PHA resulted in a greater than 2-fold increase in proliferation in the uninfected memory T-cell population. However, the presence of PHA resulted in an approximately 30% reduction in the cleavage of XTT in the HIV-1 infected population, indicating a decrease in proliferation. Conversely, αCD3/CD28 stimulation resulted in a nearly 3-fold increase in the absorbance values in both the uninfected and infected CD8+CD45RO+ memory T-cell populations.

## DISCUSSION

Despite the accumulating research (and controversy) over the nature of HIV-1 infection of CD8+ T-cells, there is still a lack of information with regard to the effects of viral infection on CD8+ T-cells and their functions. Though previous studies have examined HIV-1 infection of CD8+ T-cells, these studies were limited predominantly to **in vivo** observations and did not have the availability of sensitive techniques used currently. Many of these studies also focused on the specific and secondary cytolytic abilities of CD8+ T-cells in response to infection and failed to examine or acknowledge the role that CD8+ T-cell subsets such as memory cells play in controlling not only HIV-1 infection, but other viral infections as well. Therefore, we set out to examine the susceptibility of CD8+ T-cells and their subsets to *in vitro* HIV-1 infection using standard laboratory strains in lieu of serially passaged isolates used in the past, and to examine the effects of infection on CD8+ T-cell function.

We observed that CD8+ T-cells were able to support viral infection and replication. To determine whether or not the infection was productive, we detected HIV-1 presence by RT-PCR analysis and treated the CD8+ T-cells with AZT, a viral replication inhibitor. We found infection to be productive as the presence of the reverse transcriptase inhibitor abrogated HIV-1 production. Of interest was the observation that CD8+ T-cells supported higher levels of replication with T-cell tropic isolates and strains of HIV-1. Similarly, CD4+ T-cells also showed a preferential susceptibility to infection with T-cell tropic strains *in vitro*. An observation we found throughout our experiments were the differences in viral production and kinetics when CD8+ T-cells were isolated from numerous volunteers. This phenomenon is not restricted solely to CD8+ T-cells as it is also apparent when CD4+ T-cells and macrophages of different individuals are infected **in vitro**[30]. Our results with T-cell tropic strains and isolates were expected as both CD4+ and CD8+ T-cells express high levels of the chemokine receptor CXCR4 that is predominantly used by the T-cell tropic HIV-1 strains for binding and entry. Specifically, the T-cell tropic laboratory strain HIV_IIIB_ resulted in the greatest replication and production in the isolated CD8+ T-cell population. Interestingly, the M-tropic laboratory strain, and to a significantly lesser extent the M-tropic clinical isolate, were also able to successfully infect CD8+ T-cells. This may be explained by the presence of moderate CCR5 expression on the surface of the CD8+ T-cells.

Our experimental results have shown that CD8+ T-cells were able to support productive HIV-1 infection. Since the CXCR4 co-receptor was highly expressed on the surface of the CD8+ T-cells and the affinity of gp120 for the CD4 molecule had already been established, we examined the possibility of CD4 up-regulation on the surface of CD8+ T-cells during infection. Our results indicate that CD8+ T-cells supported approximately 10-fold lower levels of *in vitro* HIV-1 replication than what was routinely observed in CD4+ T-cells, corroborating a report that suggested that CD8+ T-cells were low producers of HIV-1 [31]. A possible explanation for this observation may be due to the low expression of the CD4 molecule on the surface of CD8+ T-cells and subsequent down-regulation of this receptor following infection. In our experiments, increases in CD4 expression by CD8+ T-cells over time in culture did not show a dependence upon infection and was not significantly observed in the *in vitro* HIV-1 infected CD8+ T-cell population. In addition, the contribution of the CD8+CD4+ T-cells in the total CD8+ T-cell population during HIV-1 infection was less than 2%. Of note was the decrease in the mean fluorescence of the CD8 cell-surface molecule in the infected population. This was most likely due to the cytopathic and anergizing effects of the culturing of CD8+ T-cells in the presence of HIV-1 or the possible down-regulation of this molecule.

Brenchley *et al.* observed that the number of HIV-1_*gag*_ DNA copies in CD4^dim^CD8^bright ^lymphocytes were 5 to 100 fold more than in the CD8+CD4- lymphocytes [32]. However, the majority of cells that were infected with HIV-1 in our *in vitro* experiments were of the CD8+CD4- phenotype as measured by intracellular HIV-1_gag_ staining. These findings suggest the possibility of an alternate portal of entry that may be used by HIV-1 to enter CD8+CD4- T-cells. The role of the CD8 cell-surface molecule in HIV-1 infection cannot be discounted as recent evidence has shown that CD4-independent HIV-1 variants exist in AIDS patients and these viruses preferentially infect CD8+ T-cells using CD8 as a receptor [10, 11].

Levels of HIV-1 replication were examined in CD8+ T-cells co-expressing CD28, CD38 or HLA-DR. These subsets were selected for study due to their significance in asymptomatic infection and disease progression. From our results, we did not see an increase in susceptibility to HIV-1 infection in the CD28+, CD38+ or HLA-DR+ CD8+ T-cell subsets. It may be argued that the observation that the positively selected populations were unable to support full HIV-1 replication was due to an inherent artifact present in the selection process with conjugated beads. However, when the memory and naïve subsets were examined later on, the positively selected memory population resulted in the higher level of HIV-1 production, thereby discounting the notion that positive selection hindered susceptibility to infection. Interestingly, these positively selected subsets showed a decrease in viral kinetics over time when compared to the negative fractions. The decrease in replication kinetics may be due to the inability of these cells to support more than a minimal level of replication. Thus, without any suitable cellular target, the virus would eventually be unable to propagate. Of note are the levels of HIV-1 replication in the CD8+CD28- subset. The number of these cells increase early on and throughout the course of infection [33, 34], suggesting that the CD8+CD28- T-cells may be involved in the control of virus replication. If the CD8+CD28- cells are indeed involved in the suppression of HIV-1 replication and, based upon our observations, show an increase in susceptibility to HIV-1 infection, then a key subset of CD8+ T-cells involved in fighting disease may be hampered or eliminated early on in the course of disease.

High levels of replication of HIV-1 were found in the CD8+CD45RO+ memory T-cell subset when compared to the CD8+CD45RA+ naïve T-cell subset. Both the CD8+CD45RO+ and CD8+CD45RA- memory T-cell populations showed significantly higher levels of replication than the CD8+CD45RO- and CD8+CD45RA+ naïve T-cell populations. However, previous research demonstrated that the CD8+CD45RA+ naïve subset was found to have a higher frequency of infection than the memory subset **in vivo**[35]. In addition, the naïve CD8+ T-cell population has also been shown to be the most susceptible to HIV-1 infection following activation-induced up-regulation of the CD4 cell-surface molecule by antibodies to CD3 and CD28 [18]. While susceptibility to productive infection was observed in the CD8+CD45RA+ naïve T-cell subset *in vitro*, our results indicate that HIV-1 replicated more efficiently and productively in the memory phenotype of CD8+ T-cells in the absence of activation-induced up-regulation of CD4 cell-surface expression. In CD4+ T-cells, it has been documented that the CD45RO+ memory population serves as the main contributor to the pool of infected T-cells **in vivo**[36, 37]. The low level of HIV-1 replication in CD8+CD45RA+ naïve T-cells may be explained by the observation in CD4+ T-cell studies that infection of naïve CD4+ T-cells by laboratory isolates of HIV-1 results in insufficient T-cell activation that is required for the reverse transcription and integration of the viral genome [38-40]. These same observations may hold true for the infection of memory and naïve CD8+ T-cells *in vitro* reported here. Work done by Wills *et al.* may partly explain the high frequency of infection of CD8+CD45RA+ naïve T-cells **in vivo**. The researchers found that the CD8+CD45RA+ T-cells are not comprised solely of naïve T-cells, but also contain a significant proportion of memory T-cells that can change from a CD45RO^high^ to CD45RA^high^ phenotype **in vivo**[41]. Thus, during the course of HIV-1 infection **in vivo**, the memory T-cell subset may play a role as the initial proviral reservoir, but may subsequently revert to the naïve T-cell phenotype that has been observed in response to various stimuli.

In order to assess the effects of HIV-1 infection on CD8+CD45RO+ memory T-cells, we examined the ability of the infected memory cells to proliferate in response to *in vitro* HIV-1 infection. It has been postulated that T-cell dysfunction could be attributed to the altered sensitivity of CD8+ (and CD4+) T-cells to various co-stimulatory signals in HIV-1 infected patients. **in vivo** results have already demonstrated the significant increase in memory T-cell numbers in acute viral infections [42, 43]. These experiments were performed in the absence of any CD4+ T-cell help. A long-standing paradigm in immunology has been the requirement for CD4+ T-cell help in the expansion and maintenance of memory CD8+ T-cells **in vivo**[44, 45]. However, evidence has demonstrated that CD4+ T-cell help was not required for the proliferation and persistence of fully functional antigen-specific memory CD8+ T-cells in the primary immune response [46]. Our findings demonstrated that HIV-1 infection of the CD8+CD45RO+ memory T-cell subset did not impair the proliferative response of these cells to the majority of the mitogens used for stimulation which may be explained by the activation state of these cells. These results may partly be explained by the low levels of HIV infection seen in the CD8+ T-cell population (Fig. **3**). If this assumption holds true, the majority of cells would be of the uninfected phenotype in the infected population. Therefore, no differences in proliferation may be due to the larger uninfected population functioning normally. Recently, Holm *et al. *demonstrated that HIV-1 virions induced CD8+ T-cells to up-regulate CD25 and HLA-DR and preferentially induced apoptosis in the CD8+CD25+HLA-DR+ T-cell subset [47]. Our isolated CD8+CD45RO+ T-cells also showed an up-regulation of HLA-DR during *in vitro* infection. However, our results also demonstrated that HIV-1 infection only minimally decreased proliferation in response to mitogenic stimulation when compared to the uninfected population. The lack of proliferation in the CD8+ memory T-cell population may be partly explained by the observations of Pantaleo *et al. *[48]. The researchers found that the CD8+HLA-DR+ subset was severely defective in their proliferative response to anti-CD28, anti-CD3, anti-CD2, PHA and PMA. From our *in vitro* results, HIV-1 infection only resulted in a noticeable decrease in proliferation in the cells stimulated with PHA. Therefore, strong mitogenic stimulation with PHA in the presence of HIV-1 resulted in the decrease in proliferation. This may have led to the triggering of an apoptotic pathway or anergy in the infected CD8+CD45RO+ memory T-cell population by an aberrant form of T-cell activation induced by PHA stimulation. More importantly, naïve cells require stimulation to proliferate and differentiate. Effector cells, on the other hand, are terminally differentiated and thus do not proliferate. Therefore, a lack of proliferation in this subset would be expected. A limitation of our studies is the delineation of the memory and naïve subsets used in the studies. Future studies will examine the phenotype of these populations and assay CD8+ T-cell function by measuring CTL ability and cytokine expression profiles.

In our *in vitro* system, CD8+CD45RO+ memory T-cells only showed an impairment of their proliferative capacity in response to HIV-1 infection in the presence of PHA stimulation. The observation that memory T-cells serve as one of the main reservoirs for the virus has profound implications as a high proportion of the expanded CD8+ T-cell population in HIV-1 infected individuals is comprised of CD45RO+ cells [49]. These memory cells have an enhanced susceptibility to cell death [50] and die upon bystander activation [51]. In addition, the CD45RO+ phenotype contributes significantly to the HIV-specific CTL response and these CD45RO+ CTLs are preferentially lost upon disease progression [52]. The effect of infection on CD8+ T-cell CTL function, chemokine and cytokine production, and the susceptibility to apoptosis could have a significant impact upon disease progression and our understanding of HIV-1 pathogenesis. The identification of a novel cellular target and viral reservoir will open up new avenues for treatment of this viral infection in the future.
